# Lipoxin suppresses inflammation via the TLR4/MyD88/NF‐κB pathway in periodontal ligament cells

**DOI:** 10.1111/odi.13250

**Published:** 2019-12-17

**Authors:** Muhanad Ali, Fang Yang, John A. Jansen, X. Frank Walboomers

**Affiliations:** ^1^ Department of Dentistry ‐ Biomaterials Radboud Institute for Molecular Life Sciences Radboud University Medical Center Nijmegen The Netherlands

**Keywords:** cytokines, inflammation, lipoxin, periodontitis, pro‐resolving

## Abstract

**Objective:**

The objective of the present study was to evaluate the anti‐inflammatory effects of lipoxin A4 (LXA4) for the treatment of periodontitis in an in vitro model.

**Methods:**

Human PDLCs were challenged with *Escherichia coli* (*E. coli*) lipopolysaccharide (LPS) to evoke an inflammatory response. This was done either in monoculture or in coculture with THP‐1, a monocytic cell line. Thereafter, cytokine expression was measured by ELISA, with or without LXA4. In addition, the effects of LXA4 were analyzed on the TLR‐MyD88‐NF‐κB (TMN)‐mediated intracellular signal pathway using immunocytochemistry.

**Results:**

In response to LPS, the level of the pro‐inflammatory cytokine tumor necrosis factor alpha increased, whereas the anti‐inflammatory cytokine interleukin‐4 decreased significantly (*p* < .05). These effects were consistently reversed when LPS‐challenged PDLCs were also treated with LXA4. The results in the coculture system were comparable to the monoculture. Immunohistochemistry and quantitative assessment confirmed the importance of the TMN signal pathway in these processes.

**Conclusion:**

These results corroborate earlier findings that PDLCs play an important role in inflammation. Moreover, LXA4 might offer new approaches for the therapeutic treatment of periodontal disease.

## 1 INTRODUCTION

Initially, gram‐negative bacteria, such as *Porphyromonas gingivalis* (*P. gingivalis*)*,* were recognized as the main factor leading to periodontitis. However, recent findings strongly suggest that not only the presence of a persistent infection, but especially the inadequate host inflammatory response to such pathogenic bacteria determine the progression of inflammation to periodontal destruction (El‐Awady et al., 2010). In this process, the periodontal ligament (PDL) is strongly implicated, as this tissue includes the cells that maintain persistent inflammatory signals in response to a bacterial insult (Van Dyke & Serhan, 2003; El‐Awady et al., 2010; Jonsson, Nebel, Bratthall, & Nilsson, 2011; Ogura et al., 1994).

Periodontal ligament cells (PDLCs) are the predominant cells of the PDL and have several important functions in tooth support, collagen production, and tissue regeneration (Lallier, Spencer, & Fowler, 2005). Recent studies showed that PDLCs also play a pivotal role in sustaining destructive immune modulators in response to inflammation promoters, such as certain components of pathogenic bacteria (El‐Awady et al., 2010). Lipopolysaccharide (LPS) is a bacterial endotoxin, which is strongly involved in the initiation and development of a host response caused by infection with gram‐negative bacteria (Chanput, Mes, Vreeburg, Savelkoul, & Wichers, 2010; Chatzivasileiou, Lux, Steinhoff, & Lang, 2013). LPS is associated with development and progression of periodontitis by activating pathogen recognition receptors (PRRs), such as toll‐like receptors (TLRs) (Trubiani et al., 2012). TLRs are transmembrane receptors which play a significant role in the progression of periodontitis (Hoshino et al., 1999). TLR4 is the principle receptor for sensing LPS from gram‐negative bacteria and is expressed in several periodontal tissue cells, including gingival fibroblasts and gingival epithelial cells (Sun, Shu, Zhang, & Wu, 2008; Wang et al., 2003). Under inflammatory conditions, the activation of TLR4 triggers myeloid differentiation primary response gene 88 (MyD88)‐dependent nuclear translocation of nuclear factor kappa B (NF‐κB) from the cytoplasm, resulting in the transcription of inflammatory genes (Ding, Zhao, Xiao, & Zhao, 2015). Host cells of the periodontium respond to LPS by synthesizing and secreting a variety of pro‐inflammatory mediators, such as tumor necrosis factor alpha (TNFα), interferon‐γ (IFN‐γ), and interleukin (IL)‐6, which thereafter play a key role in periodontal tissue breakdown (Kim & Amar, 2006). Anti‐inflammatory cytokines, including IL‐1, IL‐4, and IL‐10, are released in an attempt to resolve inflammation (Bastos et al., 2009). Therefore, pro‐ and anti‐inflammatory cytokines (for instance, the ratio between TNFα: IL‐4) are often used as an indicator of the inflammatory response and periodontitis development in patients suffering from periodontal disease (Bastos et al., 2009; Ferraz et al.., 2016).

Current treatment of periodontal disease relies on elimination of microbes by administering broad‐spectrum antibiotics, such as tetracycline, as well as preventing the recurrence of dental plaque as an adjunct to scaling and root planning (SRP) (Silverio et al., 2008). However, non‐target specificity and the increasing prevalence of drug‐resistant bacteria endanger the effectivity of this treatment. Therefore, a new form of cure based on resolution of the inflammatory process can be of interest (Gaudin, Tolar, & Peters, 2018). Lipoxins are a class of pro‐resolving mediators endogenously expressed in mammalian cells from the metabolism of arachidonic acid (AA), which act as agonists to promote resolution of inflammation (Sodin‐Semrl, Taddeo, Tseng, Varga, & Fiore, 2000). Although the potential use of the lipoxin A4 (LXA4) for the treatment of periodontal disease has been demonstrated (Pouliot, Clish, Petasis, Dyke, & Serhan, 2000), the mechanism in which LXA4 induces resolution effects has not been fully investigated.

Therefore, an in vitro coculture model is herein presented of human‐derived PDLCs and THP‐1 cells that can be manipulated to mimic the inflammatory clinical situation associated with periodontitis. The in vitro model was used to elucidate the anti‐inflammatory activity of LXA4 in LPS‐activated PDLCs either alone, or in coculture with THP‐1 cells.

## 2 MATERIAL AND METHODS

### 2.1 Reagents

Synthetic lipoxin A_4_ (LXA4) was purchased from Cayman Chemical. Dulbecco's modified eagle's medium (DMEM/F‐12), RPMI‐1640 medium, penicillin–streptomycin (PS), and trypsin–EDTA solution were all purchased from Gibco®, Thermo Fisher Scientific. Fetal bovine serum (FBS), phosphate‐buffered saline (PBS) tablets, bovine serum albumin (BSA), alamarBlue™ reagent, Pierce™ IP lysis buffer, and bicinchoninic acid (BCA) assay were all purchased from Sigma‐Aldrich. Commercially available preparations of LPS from *E. Coli* were purchased from InvivoGen. TNFα and IL‐4 ELISA kits were purchased from R&D systems. Millicell® EZ 8‐well glass slides were purchased from Merk. All cell culture flasks and plates were purchased from Greiner Bio‐one.

### 2.2 Cell sources

All experiments were done in accordance with the national guidelines for working with human materials (Dutch Federation of biomedical scientific societies, human tissue, and medical research: code of conduct for responsible use. Available at https://www.federa.org/). After informed patient consent, human PDLCs were harvested from an impacted third molar from one adult patient. PDLCs were then placed in a sterile 75 cm^2^ culture flasks with DMEM medium with 10% FBS and 1% PS (all: Gibco). Cells were cultured at 37°C in a humidified atmosphere of 95% air and 5% CO_2_, and medium was replaced every 2 to 3 days until 50% confluence was reached. PDLCs were then frozen in medium supplemented with 10% dimethyl sulfoxide (Sigma‐Aldrich). After defrosting, PDLCs at the 5th passage were used in all experiments. The human monocytic cell line, THP‐1, was purchased from the American Type Culture Collection and cultured in RPMI‐160 medium supplemented with 10% FBS and 1% PS.

### 2.3 LPS challenge and LXA4 treatment

#### 2.3.1 Monoculture

**Figure 1:**
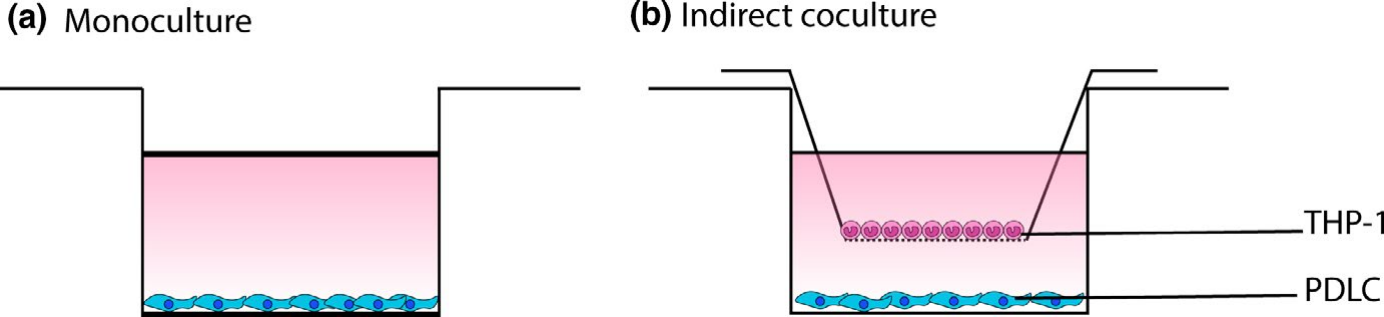
Schematic representation of the culture systems. (a) Monoculture composed of PDLCs. (b) Indirect coculture system composed of THP‐1 cells (upper compartment) and PDLCs (lower compartment) separated by 0.4 μm pore size 24‐well insert [Colour figure can be viewed at http://www.wileyonlinelibrary.com]

PDLCs were seeded at 20,000 cells/well on a 24‐well plate for 24 hr at 37°C in a humidified atmosphere of 95% air and 5% CO_2_ (Figure 1). At day 1, cells were challenged with LPS (10 µg/ml) to induce an inflammatory response for 24 hr. At day 2, cells were treated with LXA4 (100 ng/ml) for 24 hr. At day 3, cell‐free supernatant was collected and stored at −80°C for ELISA. Cells were homogenized with lysis buffer and stored at −80°C and later analyzed for protein content. In addition, parallel samples of PDLCs underwent fluorescence immunostaining and microscopic evaluation.

#### 2.3.2 Coculture

Indirect cocultures composed of PDLCs in the lower compartment and THP‐1 cells in the upper compartment were separated by 24‐well inserts of 0.4 µm pore size (Greiner bio‐one). Cells were seeded at a ratio of 1:4 (PDLC: THP‐1), which composed of a total of 2 × 10^4^ PDLCs in 800 µl that was added to the bottom of the well, and 8 × 10^4^ of THP‐1 cells in 200 µl that was added into 0.4 µm pore inserts of 24‐well transwell plates in 1:1 mixture medium (DMEM: RPMI‐160). Medium was changed on day 1, when cells were challenged with 10 µg/ml LPS. At day 2, cells were treated with LXA4 (100 ng/ml) and then incubated at 37°C in a humidified atmosphere of 95% air and 5% CO_2._ At day 3, PDLCs were homogenized with lysis buffer and stored in −80°C and later analyzed for protein content. Moreover, cell‐free supernatant was collected and stored at −80°C for ELISA. In addition, parallel samples of PDLCs underwent fluorescence immunostaining and microscopic evaluation.

### 2.4 THP‐1 differentiation

THP‐1 monocytes were examined using light microscopy, and images were taken using Leica DM 6000B light microscope. After undergoing the conditions outlined above (2.3.2), THP‐1 monocytes were subjected to visual examination and size determination by using Fiji 1.51n software (National Institute of Health). Briefly, to examine the differentiation of THP‐1 monocytes into macrophages, the diameters of 25 monocytes were measured from 4 separate photographs per condition and the average of the measurements is provided (*n* = 100).

### 2.5 Cell viability study

The alamarBlue (AB) cell viability reagent was used to confirm cell viability in the presence of LPS. PDLCs were seeded at 10,000 cells/well on a 96‐well plate for 24 hr before treatment with LPS using increasing concentrations, ranging 0, 10, or 20 µg/ml, for 24, 48, or 72 hr (*n* = 3). The cells were then washed with PBS, and AB reagent (10% of the well volume) was added to each well and incubated for 4 hr at 37°C to allow resazurin to convert to resorufin by metabolically active cells. PDLCs' viability was determined by fluorescence measurements made on Synergy HTX Multi‐Mode Reader (BioTek) using an excitation wavelength of 535 nm and an emission wavelength of 590 nm. The percentage of AB reduction was compared against untreated PDLCs (control).

### 2.6 Cytokine expression

The release of TNFα and IL‐4 expression in the culture supernatants was quantified using cell‐free supernatant after 72 hr of coculture. The ELISA was done using commercially available enzyme ELISA kits. All assays were conducted in accordance with the manufacturer's instructions (R&D Systems). Briefly, the capture antibodies were incubated overnight at room temperature (RT) on high protein‐binding 96‐well plates (Thermo Fisher Scientific). After washing (PBS + 0.05% Tween 20) and blocking (3% BSA/PBS), the cytokine standards and samples were added and incubated for 2 hr at RT, followed by incubation for 2 hr with detection antibodies. After 20‐min incubation with streptavidin‐horseradish peroxidase, 3, 3', 5, 5'‐Tetramethylbenzidine (Sigma‐Aldrich) was added and the reaction was stopped with sulfuric acid (2N). The absorbance was read using a microplate reader (Synergy HTX Multi‐Mode Reader, BioTek) at 450 nm. To normalize the amount of cytokine expressed per protein, cells were washed twice with ice‐cold PBS and homogenized with lysis buffer and the amount of protein was determined according to the BCA assay kit using a multimode spectrophotometer (BioTek) according to the manufacturer's instructions.

### 2.7 Immunostaining

To study the effect of LXA4 on LPS‐induced inflammation, cells were stained as previously reported (Diomede et al., 2017). For monoculture, PDLCs (2 × 10^3^ cells/well) were seeded in 8‐chambered glass slides (Millicell). In coculture, PDLCs were cultured in a 24‐well plate with 12‐mm glass coverslips (Deckgläser, VWR International B.V.). At day 1, the cells were challenged with 10 μg/ml of E. Coli LPS for 24 hr in a 37°C incubator with 5% CO_2_. At day 2, the cells were washed twice with PBS and then treated with 100 ng/ml LXA4 for an additional 24 hr under the same storage conditions. At day 3, the cells were fixed for 10 min at RT with 4% paraformaldehyde in PBS, pH 7.2, followed by blocking with 1% BSA. Cells were then stained with primary monoclonal antibodies anti‐human TLR4, MyD88, NF‐κB, followed by GTα (AFS94) as secondary antibody under the conditions outlined in Table 1. Before mounting for microscopic observation, samples were briefly washed in distilled water and cell nuclei were stained with 4‐6‐diamidino‐2‐phenylindole (DAPI) (1:1,000) for 10 min at 37°C (Invitrogen).

**Table 1:** List of antibodies used

Primary or secondary	Antibody	Supplier	Species	Dilution	Buffer solution	Incubation time	Temperature (°C)
Primary	TLR4	Thermo Fisher Scientific (Waltham, MA, USA)	Rabbit	1:200	BSA 1%	2 hr	23
Primary	MyD88		Rabbit	1:50	BSA 1%	2 hr	23
Primary	NF‐κB	OriGene Technologies GmbH (Herford, Germany)	Rabbit	1:200	BSA 1%	2 hr	23
Secondary	GTα	Molecular Probes (Invitrogen, Eugene, OR, USA)	Anti‐rabbit	1:200	BSA 1%	1 hr	23

### 2.8 Fluorescent microscopy

After staining and mounting, the stained slides were separated from the supports and images were acquired with a fluorescence microscope equipped with an ApoTome slide module and AxioVision 4.8 software, using 40×/1.0 objective lens (Zeiss AxioCam MRc5; Carl Zeiss Microimaging). The images were examined and photographed under the same acquisition hue, saturation, and intensity (HSI) threshold settings. The fluorescence intensity of secondary antibody was measured using Fiji 1.51n in‐built software on fluorescence micrographs of PDLCs obtained without DAPI nuclear staining. The relative fluorescence intensity compared with control was quantified for each condition under identical threshold settings of color, saturation, and brightness (*n* = 3) (Cuijpers et al.., 2015).

### 2.9 Statistical analysis

Data comparison was performed using one‐way analysis of variance (ANOVA), and pairwise multiple‐comparison test (Tukey test) was used to identify difference among the groups. All statistical analyses were performed using GraphPad Prism v.5 (GraphPad Software). Data are expressed as mean ± standard deviation (*SD*). Differences were considered to be statistically significant when *p* < .05. All the experiments were performed in triplicate (*n* = 3).

## 3 RESULTS

### 3.1 THP‐1 differentiation

**Figure 2:**
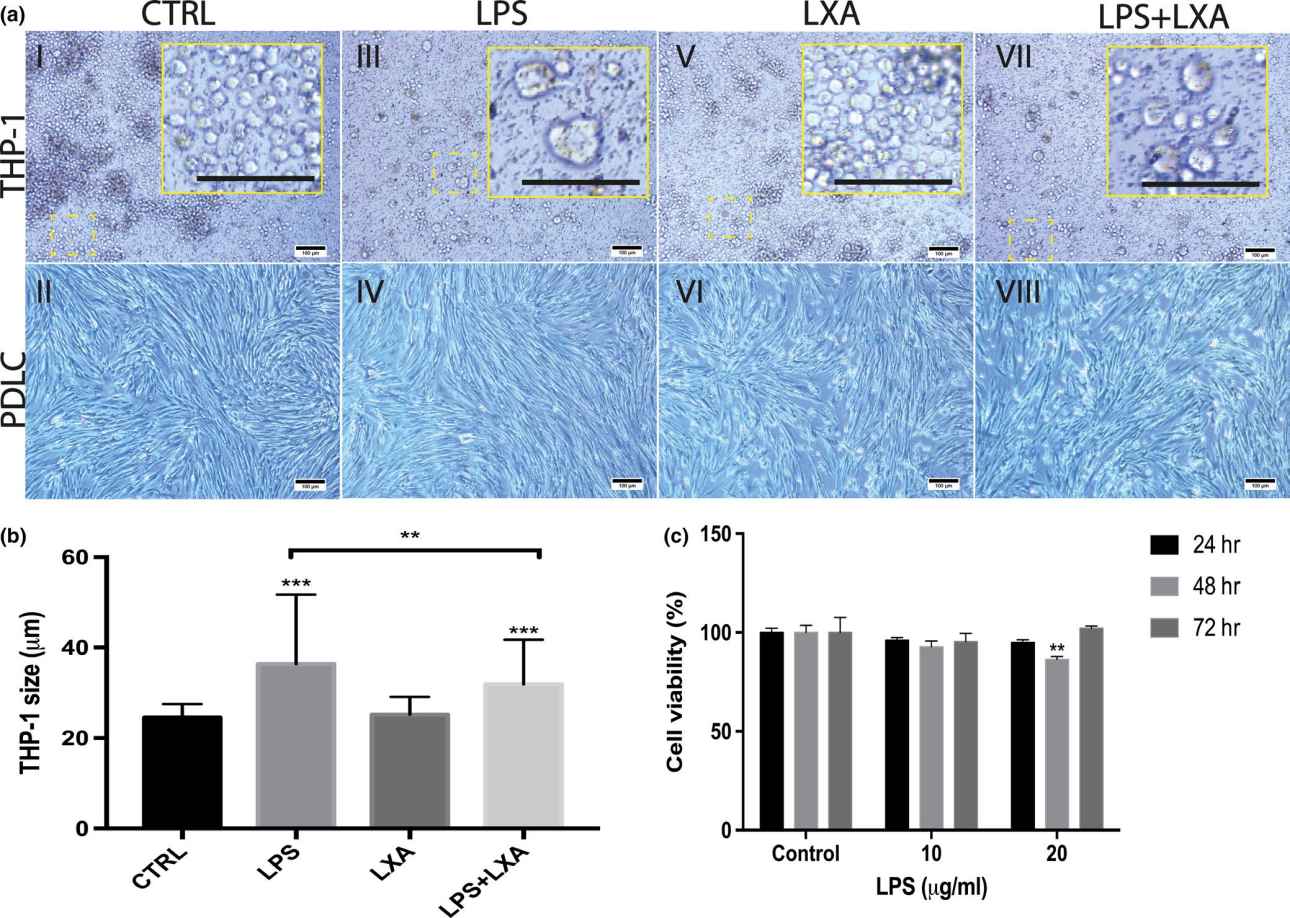
(a) Morphological examination of PDLCs and THP‐1 monocytes. (I&II) Control, (III&IV) LPS, (V&VI) LXA4, (VII&VIII) LPS + LXA4. 10x magnification. Scale bars: 100 µm. (b) Size of THP‐1 monocytes exposed to LPS, LXA, or LPS + LXA4 (*n* = 100). THP‐1 monocytes exposed to LPS resulted in a larger macrophage‐like cell population. This response was reversed in LPS‐challenged THP‐1 cells exposed to LXA4. (c) Effect of LPS on PDLCs viability (%) relative to the control. LPS induced minimal effects on cell viability of PDLCs. The significant difference is displayed relative to the control where *p < .05, **p < .01, and ***p < .001 [Colour figure can be viewed at http://www.wileyonlinelibrary.com]

Figure 2a shows that LPS challenge induced the differentiation of THP‐1 monocytes into larger macrophage‐like cells. The addition of just LXA4 alone had no apparent morphological effect by visual inspection. However, treatment of LPS challenged with LXA4 resulted in a reduction in size, albeit monocytes were still larger than the undifferentiated ones as present in the control. Figure 2b shows the quantitative analysis of monocyte/macrophage size in response to various conditions. LPS challenge resulted in monocytes with a significantly larger cell diameter compared with control (*p* < .001). Moreover, LXA4 treatment alone had no significant effect on the size of THP‐1 monocytes (*p* > .05). However, LPS + LXA4‐treated cells were significantly smaller than LPS‐challenged monocytes (*p* < .01), but still significantly larger compared to the size of the control monocytes (*p* < .001).

### 3.2 Cell viability study

Figure 2c shows the effect of LPS on PDLCs viability. PDLCs were cultured in proliferation medium supplemented with *E. Coli* LPS, ranging from 0 to 20 µg/ml for up to 72 hr. Statistical evaluation showed that there were no significant effects in response to LPS challenge with 10 µg/ml compared to control at all time points (*p* < .05). However, a significant decrease in cell viability was detected in PDLCs treated with 20 µg/ml for 48 hr compared with control (*p* < .01). No further significant differences were observed compared with control (*p* > .05).

### 3.3 Cytokine expression

**Figure 3:**
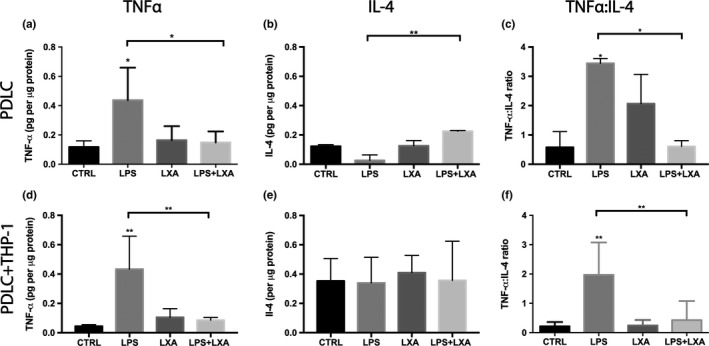
Effect of LPS stimulation and LXA4 treatment on expression of TNFα (a, d), IL‐4 (b, e), and TNFα: IL‐4 (c, f) in PDLCs (a–c), and PDLCs + THP‐1 cells in indirect coculture (d–f). LXA4 reversed LPS‐induced inflammation in monoculture and coculture. The significant difference displayed as compared to the control is *p < .05, **p < .01. Data are shown as mean ± *SD* (*n* = 3)

Figure 3 displays the amount of TNFα and IL‐4 per µg of protein in PDLCs monoculture and PDLCs/THP‐1 coculture. In monoculture (Figure 3a), after 24 hr LPS challenge, a significant increase in the expression of TNFα was observed (*p* < .05). The single addition of LXA4 had no significant effect (*p* > .05). However, treatment of LPS‐challenged PDLCs with LXA4 resulted in a significant decrease in TNFα compared to LPS (*p* < .05), to a level that was no longer significantly different from the control (*p* > .05). Figure 3b shows that IL‐4 relative expression seemed to be reduced by LPS challenge, but the difference was not statistically significant (*p* > .05). However, LXA4 treatment of LPS‐challenged PDLCs leads to a significant increase in IL‐4 compared to the LPS‐treated sample (*p* < .01). The TNFα: IL‐4 ratio showed that LPS induced a significant sixfold increase in inflammatory response compared to the control (*p* < .05), while LXA4 treatment resulted in a sixfold decrease in inflammation compared to LPS‐challenged cells in monoculture (*p* < .05) (Figure 3c).

In coculture (Figure 3d), the total amount of TNFα was significantly increased in LPS‐challenged cells compared to control cells (*p* < .01). The level of TNFα expression was also significantly lower in LPS‐challenged cells treated with LXA4 compared to LPS alone (*p* < .01). Figure 3e depicts that no significant differences existed among cells regarding total amount of IL‐4 (*p* > .05). Overall, the TNFα: IL‐4 ratio showed that LPS induced a significant ninefold increase in inflammatory response compared to the control (*p* < .01), while LXA4 treatment resulted in a fivefold decrease in inflammation compared to LPS‐challenged cells in coculture (*p* < .01) (Figure 3f).

### 3.4 Immunofluorescence staining

**Figure 4:**
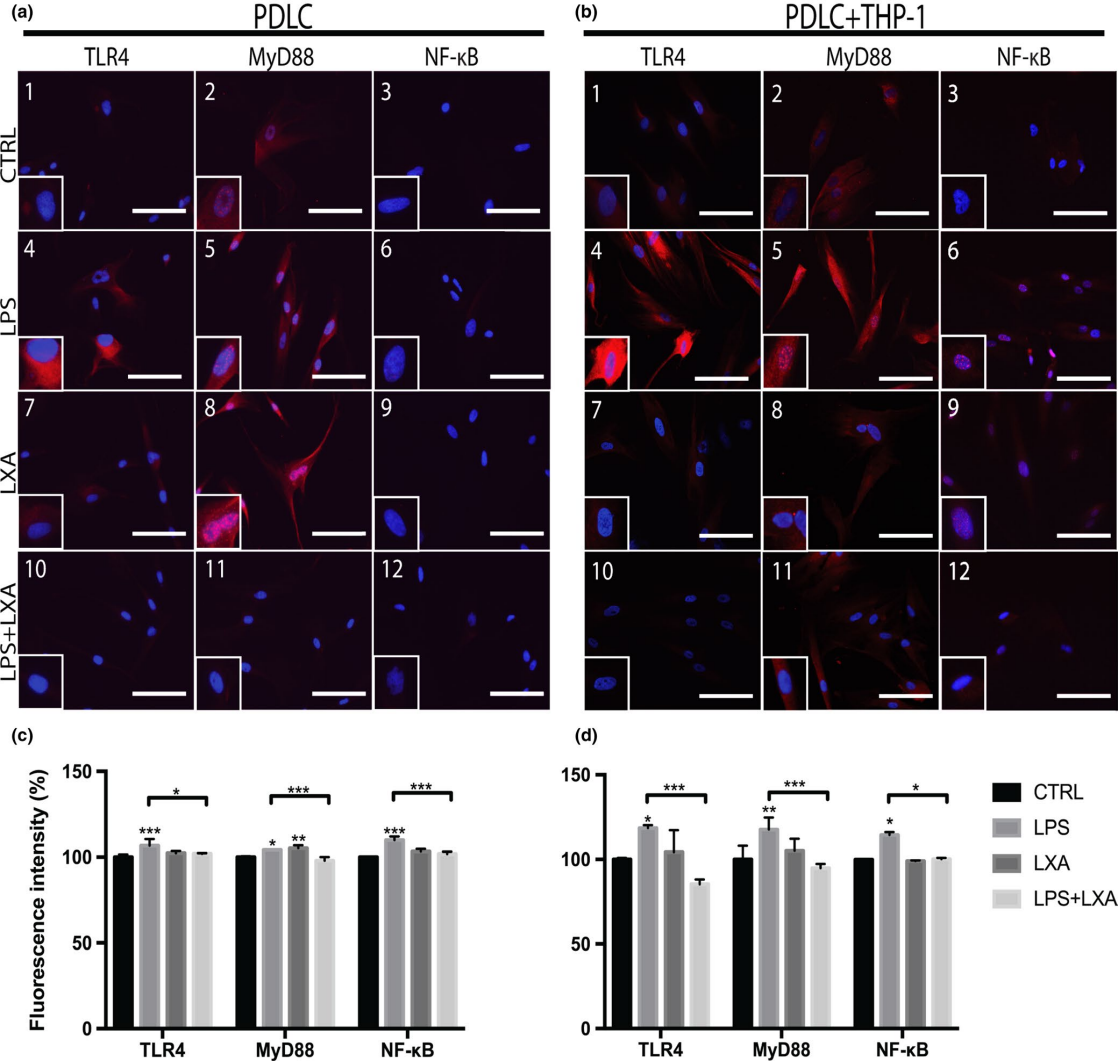
Representative immunostaining images of the expression and localization of TLR4, MyD88, and NF‐κB in human PDLCs in (a) monoculture and (b) coculture with THP‐1 monocytes. Nuclei were stained with DAPI (*blue*). Control (*red*, a and b 1–3), LPS (*red*, a and b 4–6)‐, LXA (*red*, a and b 7–9)‐, and LPS + LXA4 (*red*, a and b 10–12)‐treated PDLCs. Original magnification is 40x. Scale bar = 100 µm. Relative fluorescence intensity (%) of PDLCs in (c) monoculture or in (d) coculture with THP‐1 monocytes. The significant difference displayed as compared to the control is *p < .05, **p < .01, and ***p < .001. LXA4 inhibited the TLR4/MyD88/NF‐κB pathway in LPS‐stimulated human PDLCs. Data are shown as mean ± *SD* (*n* = 3) [Colour figure can be viewed at http://www.wileyonlinelibrary.com]

Figure 4a,b displays the effects of LXA4 on the TLR4/MyD88/NF‐κB (TMN) pathway in LPS‐challenged PDLCs or in coculture with THP‐1 monocytes. The secondary antibody staining GTα combined with nuclear staining DAPI showed minimal immunofluorescence staining in unchallenged cells for TLR4, MyD88, and NF‐κB (Figure 4a,b, 1–3). Moreover, a marked increase in immunofluorescence staining was visually observed for TLR4, MyD88, and nuclear mobilization of NF‐κB following LPS challenge (Figure 4a,b, 4–6). Conversely, immunofluorescence staining for TLR4, MyD88, and NF‐κB was reduced completely by LXA4 treatment of LPS‐challenged PDLCs (Figure 4a,b, 10–12).

Figure 4c,d displays the quantitative analysis of the relative immunofluorescence staining intensity in monoculture and coculture, respectively. Immunofluorescence staining showed that LPS treatment produced a significant increase in TLR4, MyD88, and NF‐κB compared to untreated PDLCs. In particular, quantitative analysis showed a significant increase in the nuclear translocation of NF‐κB in cells after exposure to LPS in monoculture (*p* < .001) and coculture (*p* < .05). However, these effects were completely reversed after treatment with LXA4 in PDLCs (*p* < .001) and coculture with THP‐1 monocytes (*p* < .05). Moreover, quantitative analysis also showed a significant increase in the MyD88 immunofluorescence signaling intensity in response to LXA4 treatment alone in monoculture (*p* < .01).

## 4 DISCUSSION

The present study aimed to investigate the potential role of PDLCs in inflammation and the application of LXA4 as inhibitor in this process. The obtained results provide evidence for a mechanism in which LXA4 may affect the inflammatory process by influencing the expression of pro‐ and anti‐inflammatory cytokines in LPS‐challenged cells. Further, the data suggest that this process is associated with attenuation of the signaling molecules of the intracellular TMN signaling pathway, particularly with the nuclear accumulation of the transcription factor, NF‐κB, and subsequent TNFα expression and release (József, Zouki, Petasis, Serhan, & Filep, 2002). Previous studies showed already that LXA4 acts as a main mediator of resolution of inflammation by binding to LXA4 receptor (ALXR), a G protein‐coupled receptor, expressed by many cell types, including human PDLCs (Alessandri et al., 2013; Levy et al., 2002). LXA4‐ALXR interaction then serves as an endogenous “stop signal” and inhibits recruitment of neutrophils by offsetting adhesion, chemotaxis, and transmigration across epithelial and endothelial cells (József et al., 2002). LXA4 was also shown to enhance macrophage phagocytosis by activating endogenous pathways to terminate inflammation (Serhan, 2005).

Our methodological choice was to study the immunomodulatory activity of LXA4 not only in monoculture, but also in a coculture system. THP‐1 monocytes have widely been used due to similarities in their responses to peripheral blood mononuclear cells (PBMCs). Therefore, we postulated that coculture models composed of PDLCs and THP‐1 cells would better mimic the clinical situation (Kämpfer et al., 2017). Circulating blood monocytes migrate from the vasculature to the extravascular compartment as part of the inflammatory response where they mature into tissue macrophages (Takashiba et al., 1999). Moreover, macrophages play an important part in regulating the innate and adaptive immune response to bacterial infection (Takashiba et al., 1999). Coculture models with monocytes allow the study of the immune response due to the integration of immune cells (Smith, Young, Hurlstone, & Wellbrock, 2015). Hence, the immunomodulatory effects of LXA4 were examined in this study, either in monoculture of PDLCs or in an indirect coculture system composed of PDLCs with THP‐1 monocytes.

Initially, the effect of increasing concentrations of E. coli LPS (0–20 µg/ml) was tested on the cell viability of PDLCs to exclude any cytotoxic effect. PDLCs appeared relatively viable and proliferative despite using high concentrations of LPS, with only minor negative effects on cell viability (ALBIERO et al., 2015). On the other hand, THP‐1 monocytes responded relatively strongly to LPS by differentiating into macrophage‐like cells (Trede, Tsytsykova, Chatila, Goldfeld, & Geha, 1995).

The effects of LXA4 on the release of TNFα and IL‐4 cytokines in LPS‐challenged PDLCs were studied in monoculture and coculture with THP‐1 monocytes. For this purpose, the levels of TNFα and IL‐4 were analyzed in which TNFα:IL‐4 ratio was used as predictor of inflammation (Tsurumi, Que, Ryan, Tompkins, & Rahme, 2016). The results showed that incubation of human PDLCs with LPS resulted in higher TNFα release, which corroborated with data from previous studies (Miyauchi et al., 2001; Morsczeck, Dress, & Gosau, 2012; Yamaji et al., 1995; Yoshimura et al., 1987). Moreover, a negative correlation was observed between TNFα and IL‐4 in monoculture, which was expected considering IL‐4 is a potent inhibitor of TNFα (te Velde, Huijbens, Heije, Vries, & Figdor, 1990). In addition, the results showed that a fewer amount of TNFα was secreted in cells treated with LXA4. These results are consistent with previous studies, where treatment with LXA4 resulted in a decrease in pro‐inflammatory cytokines (Gaudin et al., 2018; Pouliot et al., 2000; Reis et al., 2017).

**Figure 5:**
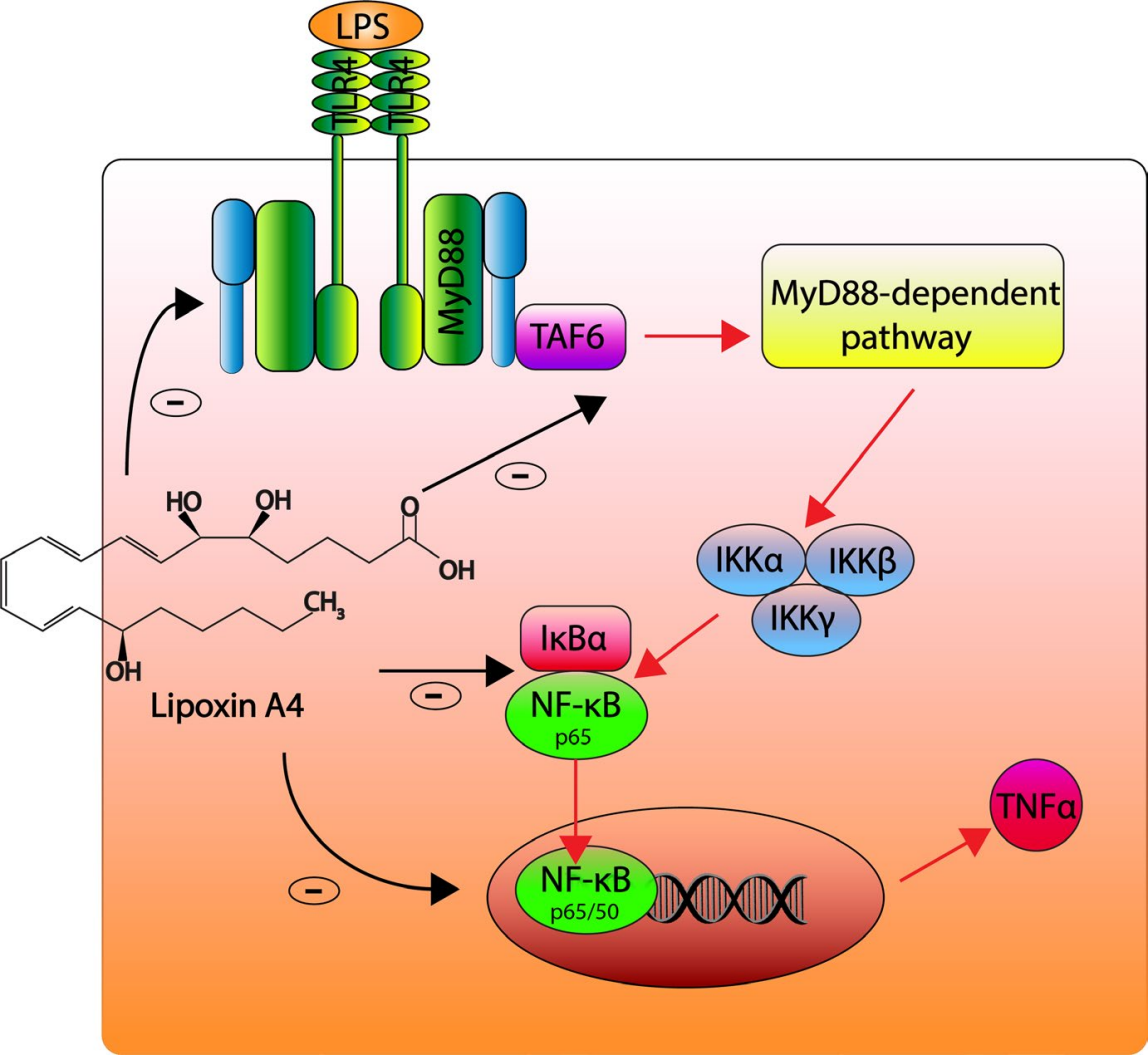
Lipoxin A4 inhibits the LPS‐induced activation of the TLR4/MyD88/NF‐κB signaling pathway in PDLCs. (Red arrows, right) LPS activates TLR4 leading to MyD88‐dependent inflammatory cascade, IκB phosphorylation, and translocation of NF‐κB from the cytosol to the nucleus, resulting in expression of TNFα. (Black arrows, left) LXA4 inhibits TLR4, MyD88, and NF‐κB signaling molecules activated by LPS [Colour figure can be viewed at http://www.wileyonlinelibrary.com]

Further, immunofluorescence staining showed an increased expression of the TLR4 receptors in response to LPS challenge. TLR4 can selectively recognize LPS and plays a key role in regulating acute inflammation (Drexler & Foxwell, 2010; Takeda & Akira, 2005). Taking into consideration that most of TLRs, with the exception of TLR3, employ the adapter protein MyD88 as signal machinery, the expression of MyD88 in LPS‐challenged PDLCs was also increased in response to LPS challenge. TLR4 activation leads to downstream MyD88‐dependent activation of kinases and subsequent phosphorylation of IκBα, which induces NF‐κB nuclear translocation from the cytosol (Lu et al., 2017). NF‐κB binds to κB sites in the promoter genes encoding pro‐inflammatory cytokines, such as TNFα (Diomede et al., 2017). Hence, over‐expression of the TMN protein induces a pro‐inflammatory cytokine inflammatory cascade as shown in Figure 5.

Immunofluorescence staining showed increased NF‐κB accumulation in the nucleus in response to LPS challenge in monoculture and coculture. However, LXA4 prevents the phosphorylation of IκBα, allowing more IκBα to bind with NF‐κB and preventing its translocation into the cell nucleus (Lu et al., 2017). As a result, LXA4 inhibited NF‐κB from binding with specific genes sequences leading to the transcription of inflammatory cytokines, such as TNFα. These observations suggest that LXA4 functions as anti‐inflammatory mediator via inhibition of the TMN pathway, which again is consistent with previous reports (Jozsef et al., 2002; Sodin‐Semrl et al., 2004).

It should also be noted that an increase in fluorescence intensity of MyD88 in the cytoplasm was observed in response to LXA4 alone, which coincided with an increase in TNFα/IL‐4 ratio in monoculture. Although the mechanism underlying these events remains unclear, an explanation for this response can be that LXA4 activates upstream signaling molecules of the TMN pathway, while other downstream molecules provide feedback on them (Lu et al., 2017). Moreover, LXA4 can activate alterative mechanisms induced by LPS stimulation as nuclear accumulation of NF‐κB was not observed (Jozsef et al., 2002). However, further in vivo studies are needed to evaluate the clinical application of LXA4 which are beyond the scope of the present study.

Overall, our findings suggest that LPS‐stimulated PDLCs and THP‐1 monocytes in coculture are a responsive in vitro system to analyze the immunomodulatory activity of LXA4. Furthermore, the preferential expression of inflammatory mediators in response to bacterial challenge provides proof for the role of PDLCs in regulating the immune response. Also, the current data strongly suggest that LXA4 exerts its anti‐inflammatory effects by inhibiting NF‐κB, which has been shown to contribute considerably to the anti‐inflammatory effects (Liu, Guan, Cai, Li, & Xiao, 2017). Hence, LXA4 may play a key role in counteracting the development of inflammation in a variety of diseases through inhibition of the TMN pathway. This can be of help in increasing the understanding of PDLCs in response to periodontal pathogens and ultimately contribute to a better therapy.

## 5 CONCLUSION

This work systematically studied the immunomodulatory effects of LXA4 in LPS‐challenged PDLCs in monoculture and in coculture with THP‐1 monocytes. It was found that LXA4 induced an anti‐inflammatory effect on LPS‐challenged PDLCs by suppressing and promoting the expression of TNFα and IL‐4, respectively. Furthermore, immunostaining revealed reduced responsiveness to LPS challenge by LXA4, in which the expressions of TLR4, MyD88, and NF‐κB in PDLCs were decreased. Our present in vitro data not only emphasize the important role of PDLCs in inflammation, but also underline that LXA4 should be implemented in the development of a therapy for periodontal disease.

## CONFLICT OF INTEREST

The authors declare that they have no conflict of interest.

## AUTHORS CONTRIBUTION

Muhanad Ali performed the experiments and wrote the article. X. Frank Walboomers, John A Jansen, and Fang Yang helped design the experiments and helped with interpretation of the data. All authors reviewed drafts of the paper.
